# Preparation of Porous Ceramsite with Ammonium Acetate as Low-Temperature Decomposition Foaming Agent and Its Sound Absorption Performance

**DOI:** 10.3390/ma12244124

**Published:** 2019-12-09

**Authors:** Huiqin Wu, Huansheng Huang, Rongjun Pan, Yeyang Chun, Ling Zhu, Kailun Nong

**Affiliations:** 1 College of Civil Engineering, Guangxi University of Science & Technology, Liuzhou 545006, China; whq6329@163.com (H.W.); huansheng_huang@163.com (H.H.); zhulingzl333@163.com (L.Z.); nongkailun@163.com (K.N.); 2 College of Civil Engineering, Guangxi University, Nanning 535004, China; chunyeyangcyy@163.com

**Keywords:** pore structure, sound absorption performance, porous ceramsite, ammonium acetate, heating rate

## Abstract

The sound absorption performance of porous ceramisite is determined by its pore structure, which is mainly governed by a foaming agent and heating rate during a foaming process. By tuning the heating rate and foaming agent concentration, ceramisite with different pore structures was prepared by using flyash, cement, quick lime, and plaster as raw materials as well as ammonium acetate as a low-temperature decomposition foaming agent in this work. The phase composition, microstructure, and sound absorption performance of the prepared porous ceramisite were investigated. Results demonstrate that the apparent porosity and the pore diameter increased with the increase of foaming agent concentration, accompanied with the broadening of the pore diameter distribution. The apparent porosity is positively correlated with heating rate until the temperature is higher than 20 °C·min^−1^, while the pore diameter is negatively correlated. The pore diameter distribution becomes narrow as a function of the heating rate. The sound absorption performance is positively correlated with the apparent porosity. An optimal pore diameter might exist, meaning diameter sizes that are larger or smaller than the optimal diameter are not conducive to the optimization of the sound absorption performance of the overall frequency band. It was determined that the curing time was not a key factor for optimizing the pore structure.

## 1. Introduction

The noise pollution caused by urban railway transit (URT) is becoming increasingly serious due to large-scale construction of urban railway transit and the increasing of train velocities. Therefore, various measures that can achieve noise abatement have been considered worldwide [1]. A sound barrier and/or track acoustic panel made of porous ceramsite, which is a kind of foaming cement-based aggregate, is quite suitable for sound absorption as both possess remarkable features, such as high sound absorption, fire-resistance, anti-seismic abilities, being weather-proof, anti-corrosiveness, a light weight, and non-toxicity [2,3]. Hence, they have been widely used to abate the noise pollution caused by URT in recent years [4,5,6]. It has been proved experimentally that the performance of the sound barrier and/or track acoustic panel is predominated by a pore structure, which is mainly determined by the pore structure of the porous ceramsite [2,7,8,9]. Therefore, it is useful to explore various approaches for preparing ceramsite using desirable pore structures.

Among the approaches that can achieve porous ceramsite, chemical foaming process is a desirable route to prepare ceramsite with tunable pore structure [10,11], in which surfactant [12,13,14], carbonate [15], carbon powder [16], SiC [17], H_2_O_2_ [18,19], aluminum powder [19], and protein [20] are traditionally used, respectively. However, although the mentioned foaming agents could achieve porous ceramsite, the foaming processes are generally performed either at high temperature or for a long duration, which makes the pore structure hard to tailor. Low-temperature decomposable ammonium salts could also be used as a foaming agent for the preparation of porous ceramsite via the foaming process. Since such foaming agents decompose at a relatively low temperature, it is much easier to tailor the pore structure when they are used for ceramsite preparation. Therefore, it is worth exploring such foaming agents.

In our previous work [2], (NH_4_)_2_CO_3_, a low-temperature decomposition ammonium salt, was used to prepare porous ceramsite. It was found that the curing duration, heating rate, and foaming agent concentration exerted remarkable influences on the pore structure [2,3]. However, although low-temperature decomposition ammonium salt was used as foaming agent in previous work, the works concerning low-temperature decomposition foaming agent are rare to our knowledge.

The thermal decomposition mechanism of the foaming agent governs the formation of pores while preparing porous ceramsite via a foaming process. However, unfortunately, it was rarely considered in previous works. To explore different foaming agents, ammonium acetate was used in the present work. The effects of the foaming agent concentration, heating rate, and curing duration on the pore structure, apparent porosity, and sound absorption performance were investigated. Due to the limitation of previous works in which the thermal decomposition of forming agent was rarely investigated, the decomposition mechanism was not available and hence it was difficult to control the pore forming process. For the decomposition mechanism of ammonium acetate in cement-based materials to be understood thoroughly, Thermogravimetry (TG) and Differential Scanning Calorimetry (DSC) techniques were used.

## 2. Experimental Section

### 2.1. Raw Materials

The raw materials used for ceramsite preparation containing fly-ash, cement, gypsum, and quick lime with a respective mass ratio of 25:5:2:1 were obtained from Ouweimu Machinery Manufacturing Co., Ltd. (Liuzhou, China). The components and proportion of fly-ash were determined according to the Methods for Chemical Analysis of Cement [21] and presented in Table 1. The used foaming agent, ammonium acetate (analytical purity grade), was purchased from Xilong Chemical Co., Ltd. (Guangzhou, China).

### 2.2. Determination of Thermal Decomposition Mechanism of Ammonium Acetate and Heat-Treatment Temperature of Ceramsite

For the decomposition mechanism of ammonium acetate to be understand thoroughly, the thermal decomposition of ammonium acetate was investigated by Thermogravimetry (TG) and Differential Scanning Calorimetry (DSC) techniques by using a simultaneous thermal analyzer (STA 449 F3, Netzsch, Selb, Germany), during which the heating rate is maintained to be 5 °C·min^−1^ in the range of 20–300 °C under a N_2_ atmosphere. To determine the thermal decomposition of ammonium acetate within ceramsite, the following samples were used for thermal analysis, respectively: (1) pure ammonium acetate powder, (2) ceramsite containing 1.83 wt.% ammonium acetate solution and raw powder with a mass ratio of 1:5 without curing, and (3) ceramsite containing 1.83 wt.% ammonium acetate solution and raw powder with a mass ratio of 1:5 after curing for 12 h.

### 2.3. Preparation of Ceramsite

In line with our previous work [2,3], 3.0 kg of raw mixture and 600.0 g of water was used for each preparation. Prior to pelletizing of the ceramsite, a weighed amount of ammonium acetate was transferred into a container with water to obtain a foaming agent solution with concentrations of 0, 0.5, 0.7, 1.0, and 1.83 wt.%, respectively. The pelletizing was performed on a ZL-500 disk granulating machine from Machinery Equipment Co., Ltd. (Zhengzhou, China), during which the foaming agent solution was sprayed to homogenously moisten the powder mixture. The pelletizing process was continued until a spherical ceramsite with the desired average diameter was achieved.

**Figure 1:**
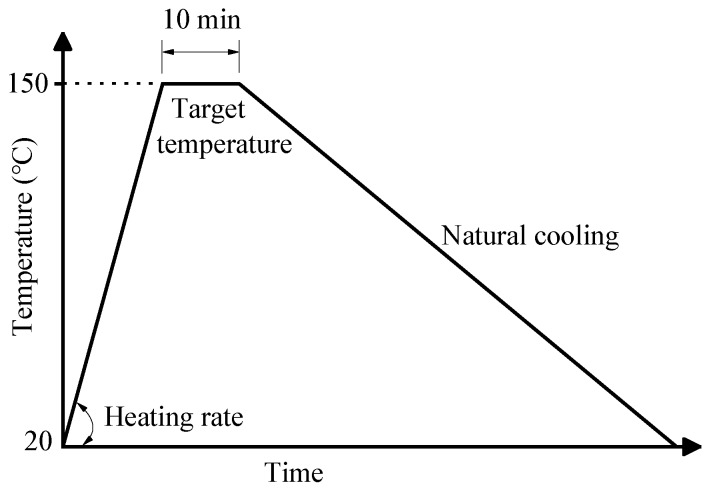
The heat treatment of the obtained ceramsite.

Prior to the heat treatment, the heating temperature and duration were determined to be 150 °C and 10 min based on the TG/DSC analytical results. The obtained ceramsite was then put into an oven for heat treatment at 150 °C, during which the heating rate was set to be 0, 3, 5, 10, 15, 20, and 30 °C/min, respectively. After being heated at 150 °C for 10 min, the samples were naturally cooled down to room temperature. (Figure 1).

### 2.4. Pore Structure Characterization and Properties Evaluation

As for sound-absorption, the apparent porosity is the dominant factor. In the present work, the apparent porosity *p* was measured by the traditional drainage method on the basis of the Archimedes principle [22]. Although some biases which might be induced by the physic basis, pore shape, accessibility of interior pores, and air within the pores could be inherent [23], mercury intrusion porosimetry (MIP) was still widely used to assess the pore size distribution in many research because of the limitation of the other characterization methods. Therefore, in the present work, the pore size distributions of the specimens were measured using an AutoPore IV9500 Automatic Mercury Porosimeter (Micromeritics, Norcross, GA, USA) with a pressure range of 0–207 MPa.

The micro-morphology of the sample was characterized by field emission scanning electron microscopy (SEM, Zeiss Sigma, Jena, Germany). The mineral composition was characterized by X-ray diffractometry (Bruker, Karlsruhe, Germany).

**Figure 2:**
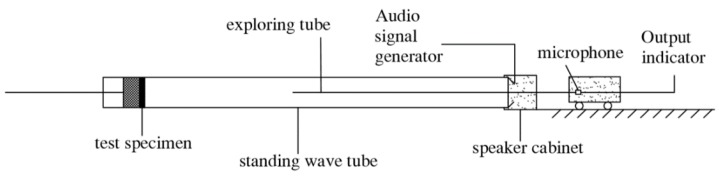
Schematic diagram of standing-wave tube method.

According to *The Measurement of the Sound Absorption Coefficient and Acoustic Impedance in the Acoustic Impedance Tube (GB/T 18696.2-2002)* [24], the sound-absorption coefficient of the specimen was tested using an AWA6128 standing-wave tube sound absorption test system from Hangzhou Aihong Instrument Co., Ltd. (Hangzhou, China). The standard cylindrical specimens with a diameter of 10 cm were made from the ceramsite. The sound waves with frequency ranging 200–2000 Hz emitted from the loudspeaker are incident on the surface of material perpendicularly in the tube, causing the sound waves to reflect back and forth in the pipeline to form a standing wave sound field (Figure 2). Thus, the maximum and minimum sound pressure will be distributed alternately along the tube axis. Hence, the values can be measured by moving the probe microphone, and therefore, the sound absorption coefficient can be evaluated according to Equation (1). When both the maximum and the minimum sound pressure are measured, Equation (2) can be used:

(1) $$\alpha =\frac{4\times S}{{\left(1+S\right)}^{2}}$$

(2) $$\alpha =\frac{4\times 10^{\Delta L/20}}{{\left(1+10^{\Delta L/20}\right)}^{2}}$$

where $S=\frac{{\left|P\right|}_{max}}{{\left|P\right|}_{min}}$, $\Delta L=L_{P_{max}}-L_{P_{min}}$; ${\left|P\right|}_{max}$ is the sound pressure maximum, ${\left|P\right|}_{max}$ is the sound pressure minimum, $L_{P_{max}}$ is the sound pressure level maximum, and $L_{P_{max}}$ is the sound pressure level minimum.

As a key mechanical performance index, the compressive strength largely dominates the practical application of ceramsite. Thus, it was evaluated by using an automatic digital compression testing machine from Tiancheng Testing Machine Manufacturing Co., Ltd. (Ji’nan, China) according to *Lightweight Aggregates and Test Method (GB17431.2-2010)* [25].

Analysis of variance (ANOVA) test was carried out for the data obtained from the samples in order to investigate the effects of the variables used in the sample preparation on the apparent porosity and cylinder compressive strength [26]. Following the determination of the significant differences of the factors, comparative analysis of the mean values of the samples were carried out in order to determine which groups showed those differences. The confidence interval for statistical tests was 95% (α = 0.05) in the study. IBM SPSS software, version 23, was used for the statistical analyses.

## 3. Results and Discussions

### 3.1. TG-DSC Thermal Analysis

**Figure 3:**
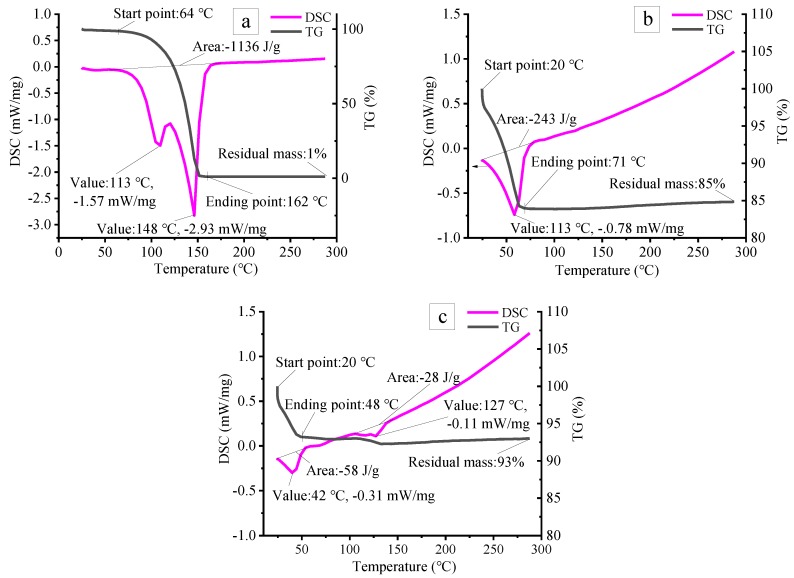
TG and DSC curves of: pure ammonium acetate (**a**) and ceramsite containing 1.83 wt.% ammonium acetate solution and raw powder with a mass ratio of 1:5 without curing (**b**) and after curing for 12 h (**c**).

As illustrated in Figure 3, for pure ammonium acetate, the TG curves shows that the initial and final decomposition temperatures are about 64 °C and 162 °C, respectively. The decomposition could be detected sharply with a mass loss of about 80% within 110–150 °C, giving gaseous N_x_O_x_ and ammonia (Figure 3a) [27]. The DSC curve further proves the above conclusion. Tow endothermic peaks at 113 °C and 148 °C are observed due to physical dissolution and chemical decomposition of ammonium acetate. Compared with pure foaming agent, the initial and final temperatures of foaming agent of the ceramsite containing ammonium acetate without curing are significantly reduced to 25 °C and 70 °C, respectively, during which the mass loss is about 16% and an endothermic peak is observed at 60.8 °C (Figure 3b). As for the sample containing ammonium acetate after curing for 12 h, the first and second weight-loss range from 25 °C to 50 °C and from 110 °C to 150 °C, respectively, in which two endothermic peaks are observed at 42 °C and 127 °C [28,29].

The thermal decomposition of ammonium acetate is affected by temperature and pH value. Without the effect of pH value, it would decompose completely below 160 °C (Figure 3a). However, for the ammonium acetate which existed in ceramsite, the mixture of cement, quicklime, flyash, gypsum, and water will provide an alkaline environment due to the hydration of cement, flyash, and quicklime, which helps to greatly reduce the initial and final temperatures. Moreover, it could be observed from Figure 3b,c that curing would exert an influence on the decomposition of the foaming agent. The longer the curing time is, the lower the final decomposition temperature becomes, implying that ammonium acetate can be used as an foaming agent to prepare porous materials at low temperature. Therefore, in the present work, the heat treatment temperature could be set to be 20–150 °C in line with the TG/DSC results.

### 3.2. Apparent Porosity

**Figure 4:**
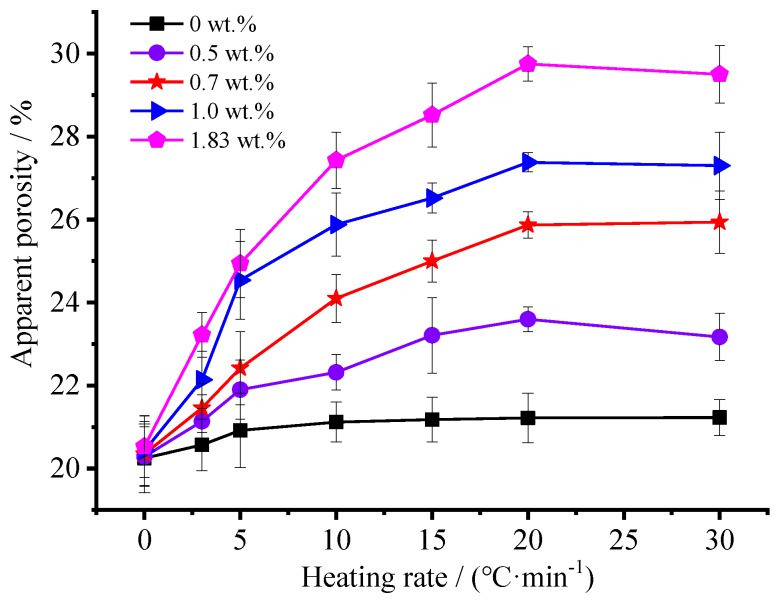
Effects of the foaming agent concentration and heating rate on the apparent porosity of the ceramsite.

Figure 4 shows the variation curve of apparent porosity as a function of heating rates and foaming agent concentration. It can be seen that the ammonium acetate concentration and heating rate exert great influence on apparent porosity, respectively. Apparently, the apparent porosity increases significantly with the increase of the foaming agent concentration. When the foaming agent was not engaged for ceramsite preparation, the apparent porosity ranged from 20.3% to 20.8%; however, conversely, the use of foaming agent would result in a remarkable increase of apparent porosity. The higher the used foaming agent concentration is, the higher the apparent porosity will be, especially for those with a foaming agent concentration higher than 1.0 wt.%. Moreover, high heating rate contributes significantly to high apparent porosity. It is worth mentioning that, a heating rate of 20 °C·min^−1^ would favor the formation of porous ceramsite with a higher apparent porosity. However, as the heating rate further increases to 30 °C·min^−1^, the apparent porosity decreases slightly.

A two-way analysis of variance (ANOVA) test was carried out for the data obtained from the samples and the results are shown in Table 2. The ANOVA findings on the effect of the factors ‘heating rate’ and ‘foaming agent concentration’ on the apparent porosity of the ceramsite listed in Table 2 showed statistical significance (*p*-value = 0% < 5%). The statistical model explained 96% of the variability observed, 40% was attributable to the heating rate, 43% to the foaming agent concentration, and 13% to the interaction between them [26].

Ideally, the elastic modulus and surface ultimate tension of matrix are assumed to be fixed values [30,31]. When ammonium acetate was introduced into the matrix, the decomposition of the foaming agent would result in an increase of pressure and hence numerous bubbles appeared within the matrix. The higher the foaming agent concentration is, the more the amount of gas derived from the decomposition of foaming agent becomes, hence leading to more bubbles forming within the ceramsite, resulting in thinner walls between neighboring bubbles. According to the Young–Laplace formula [32,33,34], the formation of pore in cement-based composite is just the process of balancing the paste pressure, that is the pore internal pressure and cement paste surface tensile force of three interactions with each other. Therefore, the pressure gradient caused by the difference of the radius of the curvature between two neighboring bubbles which promotes the radius tends to be the same. When the pressure gradient is large enough to overcome the surface tensile force, the air bubbles’ breakage will occur, resulting in connected or open pores within the ceramsite [33]. That is the reason why a higher foaming agent concentration resulted in higher apparent porosity.

Furthermore, the formation of a porous structure depends on the competition of gas bubbles and polycondensation reactions which result in hardening [35]. For a single component system, the relationship of the pressure and temperature could be evaluated by the Clapeyron Equation when two phases of solid and gas are in equilibrium [36]:

(3) $$d_{P}/d_{T}={\Delta H}_{m}/\left(T\cdot {\Delta V}_{m}\right)$$

When temperature rises from T to $(T\;+\;d_{T}$), the pressure should increase from P to $(P\;+\;d_{P}$) accordingly to maintain the balance, which in turn results in an enlarged volume of bubbles and hence thinner walls between neighboring bubbles. When the pressure gradient is large enough to overcome the surface tensile force, the air bubbles’ breakage will occur, resulting in connected or open pores within the ceramsite [33]. As a higher heating rate would result in a higher *d_T_*, the bubbles would be subject to a higher pressure gradient according to the Clapeyron Equation. As a result, the open pores extend inward continuously, resulting in higher apparent porosity. When the heating rate is low, the decomposition of foaming agent is low, producing a small amount of gas, while the small pressure gradient makes the bubbles grow slightly. At the same time, the heat distribution uniformity results in a uniform distribution of thermal stress, and the equilibrium of surface tension and pressure inside the bubble will be established easily, hence more closed pores because of more trapped gas inside the ceramsite. However, a higher heating rate will not only result in a higher *d_P_* and a more rapid decomposition of foaming agent, but will also accelerate the polycondensation reactions greatly. Therefore, an equilibrium between the two influence factors would reach resultantly [35]. With the heating rate further increases, the polycondensation reactions will be dominant. Hence, the formed pores will shrink inwards due to the hydration of the matrix surrounding the pores, which results in a slight decrease of apparent porosity.

### 3.3. Microstructure of the Obtained Ceramsite

#### 3.3.1. Effect of Foaming Agent Concentration on the Micromorphology of the Ceramsite

**Figure 5:**
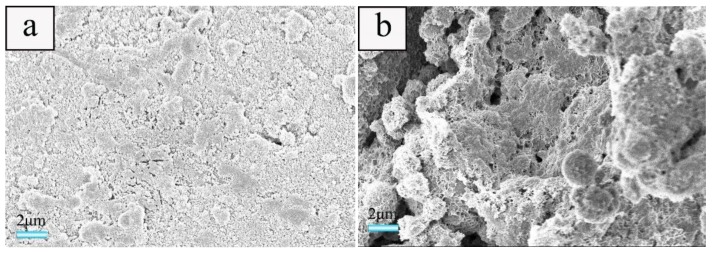

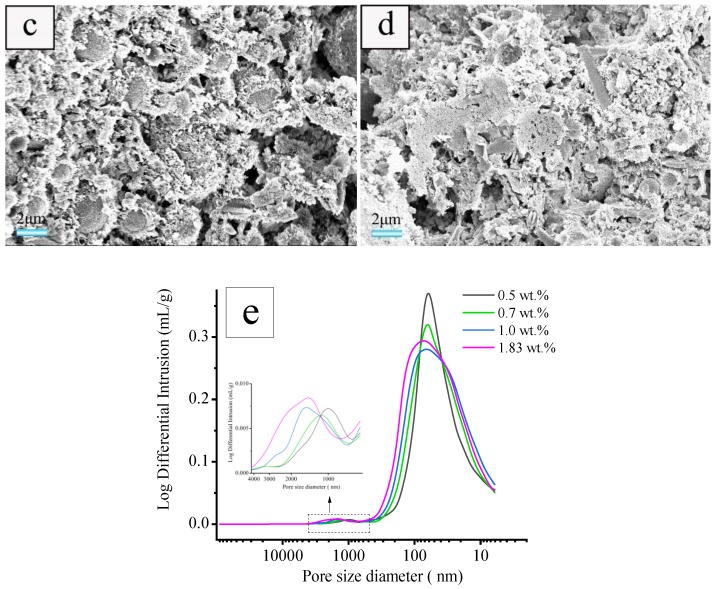
SEM images of cross-section of the samples obtained at a heating rate of 20 °C·min^−1^ with various foaming agent concentrations of: (**a**) 0.5, (**b**) 0.7, (**c**) 1.0, and (**d**) 1.83 wt.% as well as the pore diameter distributions of the samples (**e**).

In order to study the effect of foaming agent concentration on the microstructure of the ceramsite, the samples treated at a heating rate of 20 °C·min^−1^ with various foaming agent concentrations were selected as a probe for SEM and mercury intrusion porosimetry measurements. The results indicate that when the foaming agent concentration is 0.5 wt.%, only a few pores could be seen (Figure 5a). The pore diameter distributions located at about 5–700 nm and 800–4000 nm were relatively narrow, which is consistent with traditional porous concrete [35]. Moreover, for the latter, it was dominated by the pores with a diameter ranging from 800–200 nm (the black curve in Figure 5e). With the increase of ammonium acetate concentration, increasing pores could be seen obviously, together with larger pore diameter and wider pore diameter distribution. With the concentration reaching 1.83 wt.%, a porous structure could be obtained, the pore diameter become bigger, and the diameter distributions at 5–700 nm and 800–4000 nm become much wider in comparison with those of the samples obtained using lover foaming agent concentrations, which further confirms the result of the apparent porosity in Section 3.1.

As illustrated in Section 3.2, the effect of ammonium acetate on the pores is attributed to the change of the internal pressure in the bubbles caused by the decomposition of ammonium acetate. As demonstrated that the particle packing, evaporation of water, and decomposition of foaming agent would result in the formation of pores, respectively. At a low foaming agent concentration, the particle packing and evaporation of water would dominate pore formation. With the increase of ammonium acetate concentration, a larger amount of bubbles is provided in comparison with low foaming agent concentration. The differences in the radius of curvature between two neighboring bubbles promotes the radius being the same, that is to say, it favors the integration of bubbles [35], which results in bigger and open pores.

#### 3.3.2. Effect of Heating Rate on Microstructure of the Ceramsite

**Figure 6:**
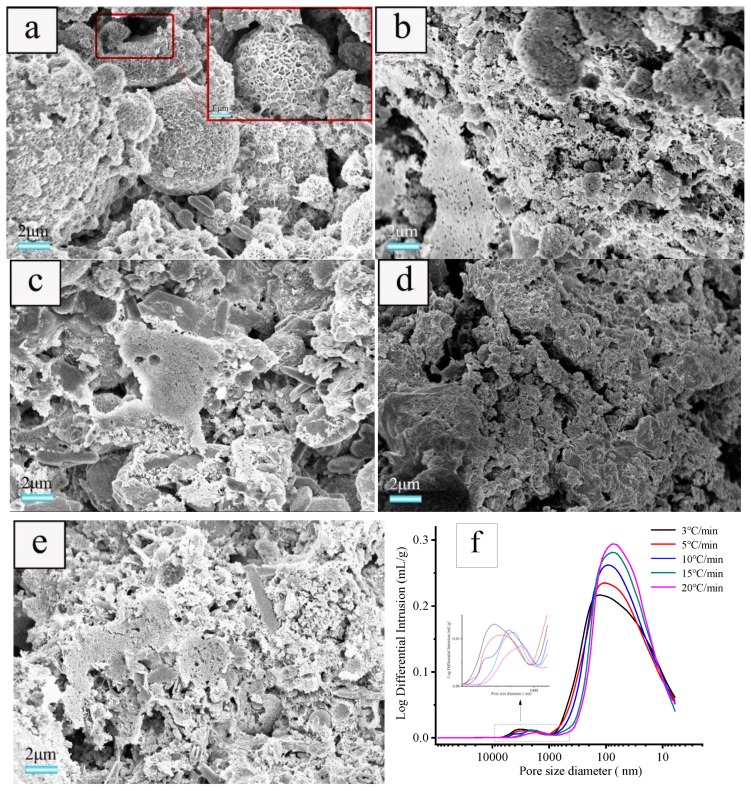
SEM images of cross-section of the samples obtained with foaming agent concentration of 1.83 wt.% and heated at heating rates of: (**a**) 3, (**b**) 5, (**c**) 10, (**d**)15, and (**e**) 20 °C·min^−1^ as well as the pore diameter distribution of the samples (**f**).

The samples prepared using a foaming agent concentration of 1.83 wt.% and heat-treated at various heating rates of 3, 5, 10, 15, and 20 °C·min^−1^ were selected for SEM and mercury intrusion porosimetry characterizations to evaluate the effect of the heating rate on the microstructure of the sample. As illustrated in Figure 6, the heating rate has a significant effect on the pore structure of ceramsite. With a heating rate of 3 °C·min^−1^, flocculent hydration products of fly ash are observed attaching to the surface (Figure 6a), which indicates that the hydration degree of fly ash is relatively low [37]. The pore diameter distributions located at ca. 5–700 nm and 800–4000 nm are wide and the pores with big diameter are dominant. When the heating rate increases from 3 to 20 °C·min^−1^, the amount of formed pore increases, while the pores with smaller diameter will predominate and the pore diameter distribution becomes narrower, especially for the sample prepared with a heating rate of 20 °C·min^−1^ (Figure 6f).

A one-way ANOVA test was run to study the effect of the heating rate on the cylinder compressive. As shown in Table 3, the univariate analysis findings on the effect of the factor heating rate on the cylinder compressive strength of the ceramsite showed statistical significance (0% < *p*-value = 0% < 5%) [26].

**Figure 7:**
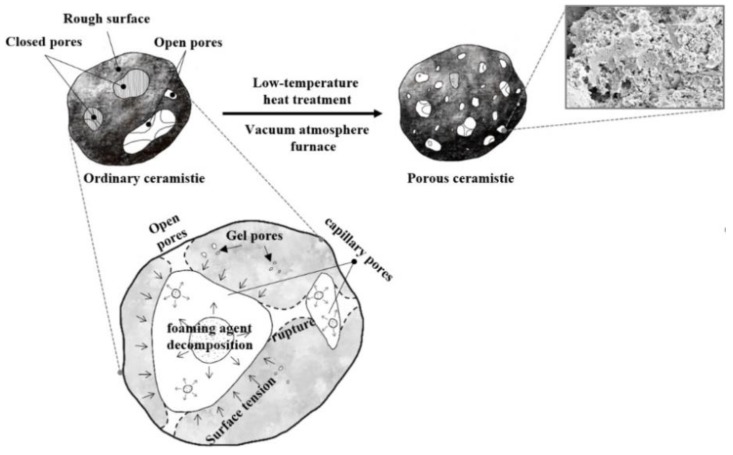
Diagram for the formation mechanism of pores.

The heating rate determines not only the change of thermal stress caused by heat conduction effect and the growth rate of bubble internal pressure, but also the heating duration and polycondensation reactions [35]. When the heating rate is relatively low, a long heating duration enables excessive free water in the capillary to evaporate [28], the heat distribution uniformity results in uniform distribution of thermal stress, and the equilibrium of surface tension and pressure inside the bubble is established easily, hence there are fewer open pores because there is more trapped gas inside the ceramsite [33,38]. At the same time, hydration products like C–S–H gels were further stripped down, which made the structure of hydration products more densified [39]. With the heating rate increase, the decomposition rate of foaming agent and the polycondensation of reactions became rapid. Before the equilibrium between them was reached, the pressure gradient external and internal bubbles would overcome the surface tension quickly according to Clapeyron Equation, and hence more connected and open pores within the ceramsite were produced. However, as the heating rate further increases, the polycondensation reactions will predominate greatly [35]. Hence, the formed pores will shrink inwards due to the hydration of the matrix surrounding the pores, leading to a slight decrease of the pore diameter and a narrower diameter distribution [40], as schematically shown in Figure 7.

**Figure 8:**
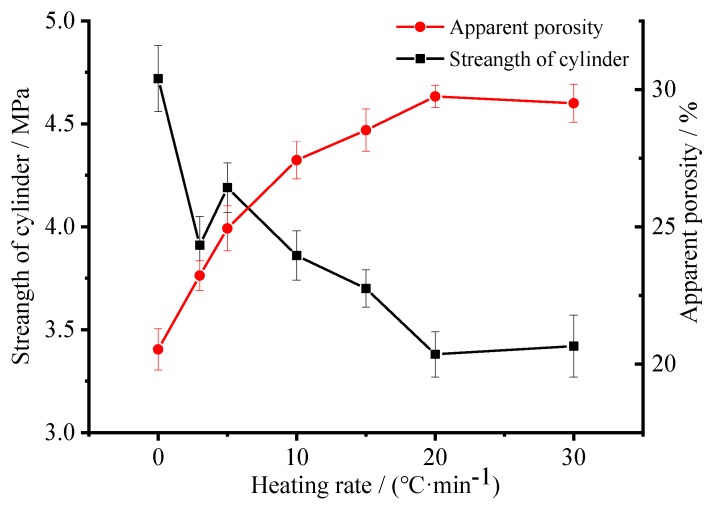
Variation of cylinder compressive strength and apparent porosity as a function of heating rate of the ceramstie obtained with foaming agent concentration of 1.83 wt.%.

It has been demonstrated that pore size and porosity is usually vital to the mechanical performance [41]. Therefore, the variation of cylinder compressive strength and apparent porosity as a function of heating rate of the ceramsite obtained with a foaming agent concentration of 1.83 wt.% was conducted to further confirm the microstructure. As shown in Figure 8, with the heating rate increasing from 0 to 20 °C/min, the apparent porosity possesses an increasing trend; however, when the heating rate further increases to 30 °C/min, it decreases slightly because of the pores’ shrinkage caused by the hydration of the matrix surrounding the pores [35,40]. As for the cylinder compressive strength, with the heating rate increasing from 0 to 30 °C/min, it by and large decreases as a function of the heating rate, indicating that a higher porosity results in a lower compressive strength [41]. As for the obtained porous ceramsite, the compressive strength is ≥3.0 MPa, which meets the requirement of sound absorbing materials as a cylinder compressive strength of ≥2.0 Mpa is needed for a sound absorbing panel or sound barrier [42].

### 3.4. XRD Analysis

**Figure 9:**
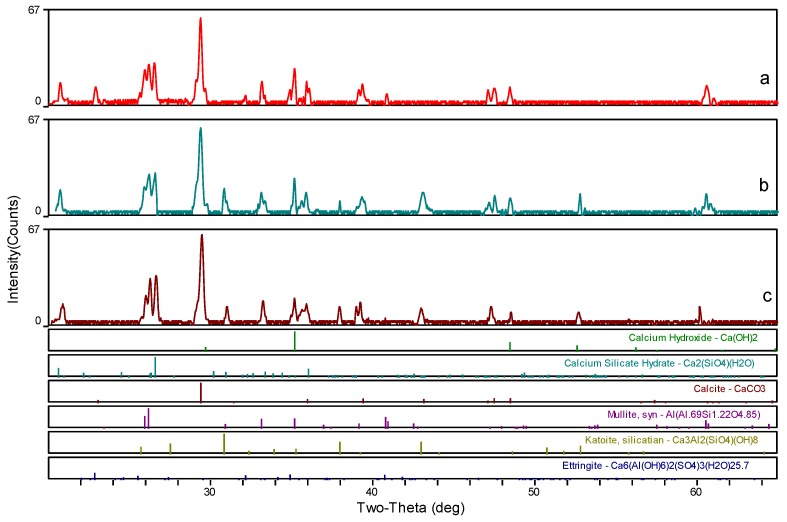
XRD patterns of the ceramsite: (**a**) without foaming agent or heat-treatment, (**b**) with heat-treatment, and (**c**) with both foaming agent and heat-treatment.

To determine the mineralogical characteristics of the samples, three ceramsite samples were used for XRD characterization: (a) without foaming agent or heat-treatment, (b) with heat-treatment, and (c) with both foaming agent and heat-treatment. As shown in Figure 9, the main components of the three samples are calcium, carbonate, mullite, and hydration products of C–S–H and C–H, respectively. The characteristic reflection peak of ettringite derived from hydration is weakened after being heat-treated, which is attributed to the poor thermal stability of ettringite [43]. At the same time, a new phase, Katoite (a kind of calcium aluminosilicate hydrate product) was observed after heat treatment because it has better thermal stability than ettringite [39]. Moreover, the diffraction peak of C–S–H was maintained in almost all the samples, implying that the bond between CaO and silicate cannot be destroyed at such temperature [29,44]. Therefore, it further confirmed that a heat-treatment temperature of 150 °C is harmless for the cement-based ceramsite materials.

### 3.5. Sound Absorption Performance

#### 3.5.1. Effect of Foaming Agent Concentration on Sound Absorption Performance

**Figure 10:**
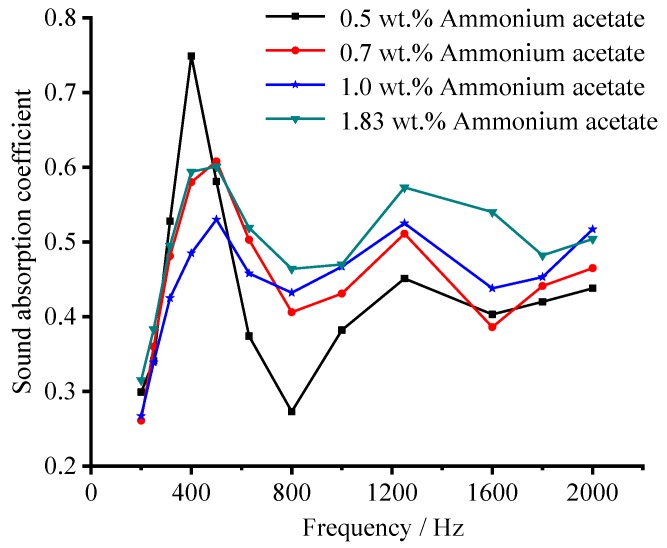
Effect of foaming agent concentration on the sound-absorption coefficient.

Figure 10 shows the sound absorption coefficient of the sample with different ammonium acetate concentrations in the frequency range of 200 to 2000 Hz. When the concentration of ammonium acetate is 0.5 wt.%, its sound absorption performance in frequency range of 200–500 Hz is excellent, while poor in the rest frequency range. As the concentration of ammonium acetate solution increases, the sound-absorption coefficient located at 200–630 Hz decreases, while enhanced in the range of 630–2000 Hz. When the concentration of ammonium acetate solution is 1.83 wt.%, the sound absorption performance is optimal in the whole frequency range, with a sound-absorption coefficient variation of 0.4–0.60 in the range of 200–630 Hz and 0.47–0.57 in the range of 630–2000 Hz. This is due to the increase of pore size and porosity. It is also proven that ammonium acetate is also an effective foaming agent for the regulation of pore structure of porous ceramsite.

As illustrated previously, a low foaming agent concentration results in low apparent porosity and small pore size. Hence, such small pores whose size is close to being nano-sized would favor the absorption of low-frequency noise because of strong scattering, reflection, and refraction of the acoustic energy [45,46]. However, ceramsite with a small pore size and low porosity means a relatively dense structure, meaning the medium-high frequency sound wave will find it much more difficult to penetrate the porous material, resulting in poor acoustic consumption [6]. As the foaming agent concentration increases, the sound-absorption performance in low frequency (200–630 Hz) seems to be more sensitive to the changing of the pore size. This may be because the sound wave with low frequency can more easily penetrate the porous material than that with a high frequency, resulting in a poor sound absorption in low frequency [47,48].

#### 3.5.2. Effect of Heating Rate on Sound Absorption Performance

**Figure 11:**
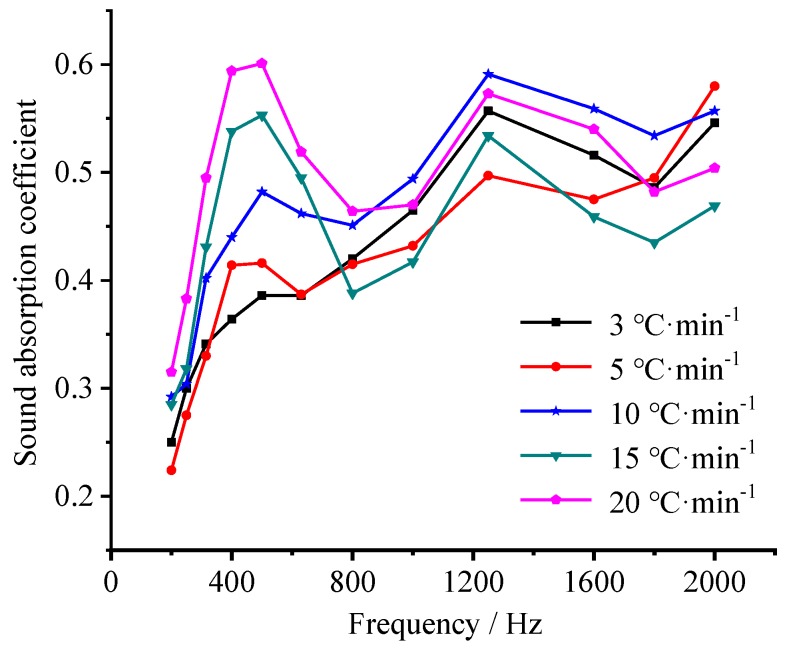
Effect of heating rate on the sound-absorption coefficient of the samples prepared with a foaming agent concentration of 1.83 wt.%.

Figure 11 shows the sound-absorption coefficient in a frequency range of 200–2000 Hz of the samples prepared at different heating rates. Obviously, the heating rate affects the sound-absorption performance of the samples significantly. At a heating rate of 3 °C·min^−1^, a strong sound-absorption could be detected only around 1200 Hz. As the heating rate increases, besides strong sound-absorption in the range of 800–2000 Hz, the sound-absorption in 200–630 Hz increases significantly, providing a sound-absorption coefficient peak gradually within this frequency range. When the heating rate is 20 °C·min^−1^, an optimal sound-absorption performance during the whole frequency range appeared, providing a sound-absorption coefficient of 0.32–0.60 in 200–800 Hz and 0.47–0.57 in 800–2000 Hz, respectively. This is consistent with the effect of foaming agent concentration on the sound absorption performance [47,48].

## 4. Conclusions

By exploring the thermal decomposition mechanism of ammonium acetate, a porous ceramsite was prepared using fly-ash, cement, gypsum, quick lime, and ammonium acetate via a foaming process in this study. The effects of foaming agent concentration and heating rate on the micro-structure of the ceramsite and its sound absorption performance were studied. The results show that the ammonium acetate can be used as foaming agent for preparation of porous materials at low temperature. The apparent porosity increases significantly with the increase of the foaming agent concentration—when foaming agent was not engaged for ceramsite preparation, the apparent porosity ranged from 20.3 to 20.8; however, conversely, the use of foaming agent would result in a remarkable increase of the apparent porosity. The higher the used foaming agent concentration is, the higher the apparent porosity will be. Moreover, a high heating rate contributes significantly to a high apparent porosity. It is worth mentioning that a heating rate of 20 °C·min^−1^ would favor the formation of a porous ceramsite with a higher apparent porosity. As the heating rate further increases to 30 °C·min^−1^, the apparent porosity would decrease slightly. Heat-treatment helps to provide Katoite, derived from the decomposition of Ettringite. The sound absorption performance is positively correlated with the apparent porosity. A too large or too small pore size is not conducive to a good sound-absorption performance.

## Figures and Tables

**Table 1:** Chemical composition of fly ash.

Component	SiO_2_	Al_2_O_3_	Fe_2_O_3_	CaO	MgO	SO_3_	TiO_2_	K_2_O	Na_2_O	LOI
Proportion (wt.%)	57.8	26.5	5.8	4.1	1.7	0.6	0.3	0.8	0.2	2.2

**Table 2:** Results of two-way ANOVA.

Source of Variation	Type III Sum of Squares	Df	Mean Squares	*F*	*p*-Value
Corrected model	1394.569 ^a^	34	41.017	92.475	0.00 (5.8382 × 10^−80^)
Intercept	97794.425	1	97794.425	220484.157	0.00 (1.0034 × 10^−225^)
heating rate	581.440	6	96.907	218.483	0.00 (1.7185 × 10^−68^)
foaming agent concentration	624.861	4	156.215	352.198	0.00 (5.4974 × 10^−72^)
Interaction between heating rate and foaming agent concentration	188.268	24	7.845	17.686	0.00 (2.2857 × 10^−31^)
Error	62.096	140	0.444		
Total	99251.090	175			
Corrected total	1456.666	174			

Df: degree of freedom. ^a^ R^2^ = 0.957 (corrected R^2^ = 0.947).

**Table 3:** Results of one-way ANOVA.

Source of Variation	Type III Sum of Squares	Df	Mean Squares	*F*	*p*-Value
Corrected model	6.526 ^a^	6	1.088	64.067	0.00 (4.6559 × 10^−15^)
Intercept	528.146	1	528.146	31111.877	0.00 (3.3763 × 10^−44^)
Heating rate	6.526	6	1.088	64.067	0.00 (4.6559 × 10^−15^)
Error	0.475	28	0.017		
Total	535.147	35			
Corrected total	7.001	34			

Df: degrees of freedom. ^a^ R^2^ = 0.932 (corrected R^2^ = 0.918)
